# Why a d-β-hydroxybutyrate monoester?

**DOI:** 10.1042/BST20190240

**Published:** 2020-02-25

**Authors:** Adrian Soto-Mota, Nicholas G. Norwitz, Kieran Clarke

**Affiliations:** Department of Physiology, Anatomy and Genetics, University of Oxford, Oxford, U.K.

**Keywords:** ketone bodies, Ketone monoester, ketosis

## Abstract

Much of the world's prominent and burdensome chronic diseases, such as diabetes, Alzheimer's, and heart disease, are caused by impaired metabolism. By acting as both an efficient fuel and a powerful signalling molecule, the natural ketone body, d-β-hydroxybutyrate (βHB), may help circumvent the metabolic malfunctions that aggravate some diseases. Historically, dietary interventions that elevate βHB production by the liver, such as high-fat diets and partial starvation, have been used to treat chronic disease with varying degrees of success, owing to the potential downsides of such diets. The recent development of an ingestible βHB monoester provides a new tool to quickly and accurately raise blood ketone concentration, opening a myriad of potential health applications. The βHB monoester is a salt-free βHB precursor that yields only the biologically active d-isoform of the metabolite, the pharmacokinetics of which have been studied, as has safety for human consumption in athletes and healthy volunteers. This review describes fundamental concepts of endogenous and exogenous ketone body metabolism, the differences between the βHB monoester and other exogenous ketones and summarises the disease-specific biochemical and physiological rationales behind its clinical use in diabetes, neurodegenerative diseases, heart failure, sepsis related muscle atrophy, migraine, and epilepsy. We also address the limitations of using the βHB monoester as an adjunctive nutritional therapy and areas of uncertainty that could guide future research.

## Introduction

The human brain consumes between 100 and 120 grams of glucose daily. As 1.75 grams of muscle protein must be broken down to produce 1 gram of glucose, lean tissue mass would quickly atrophy in order to feed the glucose-deprived brain in early starvation [1], should no other adaptation take place. To decrease muscle atrophy, fuel the brain and allay rapid death in starvation, d-β-hydroxybutyrate and acetoacetate are produced from our plentiful fat stores in a process named, ketogenesis [2].

### Physiological effects of ketone bodies

Having a higher H:C ratio than pyruvate (2 vs. 1.3), ketone bodies are more reduced, so can yield more free energy per mol of oxygen to fuel ATP production [3] and produce fewer reactive oxygen species (ROS) than glucose and fatty acids [4]. More than an efficient fuel, ketones regulate their own production by inhibiting lipolysis, spare glycogen and direct fuel oxidation in different tissues [5]. βHB acts as a signalling molecule in different tissues to co-ordinate a survival response during starvation; it increases histone acetylation, inducing the expression of genes that suppress oxidative stress [6], diminishes inflammation by blocking the NLRP3 inflammasome [7] and reduces sympathetic nervous system activity and total energy expenditure by inhibiting short-chain fatty acid signalling through GPR41 [8]. Finally, the βHB molecule is a histone modifier and directly regulates gene expression [9].

### Production of ketone bodies

**Figure 1.**
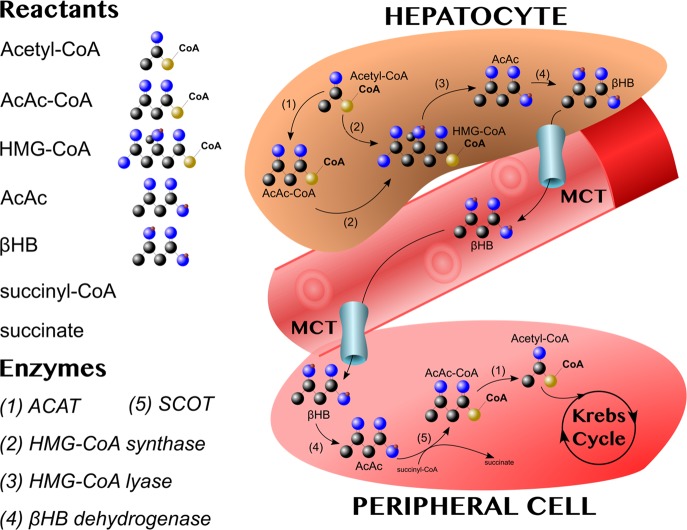
In hepatocytes, acetyl-coenzyme A acetyltransferase (ACAT: 1) combines two acetyl-CoA molecules into acetoacetyl-CoA (AcAc-CoA). AcAc-CoA is combined with another acetyl-CoA by HMG-CoA synthase (2) to form 3-hydroxymethylglutaryl-CoA (HMG-CoA). HMG-CoA lyase (3) cleaves HMG-CoA, releasing acetyl-CoA and the ketone body, acetoacetate (AcAc). AcAc can then be reduced to βHB by βHB dehydrogenase (4). βHB, the main transport ketone, exits hepatocytes via monocarboxylate transporters (MCT) and travels through the circulation to peripheral tissues. Once there, βHB is oxidised back into AcAc by βHB dehydrogenase (4). In the rate-limiting step of ketolysis, succinyl-CoA-3-oxaloacid CoA transferase (SCOT) (5) converts AcAc and succinyl-CoA into AcAc-CoA and succinate. AcAc-CoA is then cleaved by ACAT (1) to yield two molecules of acetyl-CoA that can enter the Krebs cycle.

In fasting, the combination of low insulin with high cortisol and glucagon stimulates adipocytes to release non-esterified fatty acids into the bloodstream. Fatty acids are taken up by hepatocytes, which is where ketogenesis takes place [10]. Acetyl-coenzyme A acetyltransferases (ACAT) are ubiquitous enzymes that catalyse the formation of acetoacetyl-CoA (AcAc-CoA) from two molecules of acetyl-CoA, and vice versa (Figure 1). AcAc-CoA is converted to 3-hydroxymethylglutaryl-CoA (HMG-CoA) by HMG-CoA synthase. HMG-CoA lyase then cleaves HMG-CoA, releasing acetyl-CoA and the ketone body, acetoacetate (AcAc) [5]. From this point, AcAc can have one of three fates: enter the bloodstream through the monocarboxylate transporters (MCT) 1–4 [11], spontaneously decarboxylate into CO_2_ and acetone (both fat-soluble molecules that will diffuse out of hepatocytes and exit the body through ventilation), or be reduced by βHB dehydrogenase into βHB, which exits through the MCT [12].

### Oxidation of ketone bodies

At target tissues, βHB is oxidised into AcAc in the reverse reaction catalysed by βHB dehydrogenase. Next, in the rate-limiting step of ketolysis, succinyl-CoA-3-oxaloacid CoA transferase (SCOT) converts AcAc and succinyl-CoA into AcAc-CoA and succinate. AcAc-CoA is then cleaved by ACAT to yield two molecules of Acetyl-CoA that can enter the Krebs cycle. As an economic survival adaptation, hepatocytes do not express the rate-limiting enzyme, SCOT, and are thus incapable of metabolising the ketone bodies they produce [13]. Also, in contrast with the conversion of glucose to pyruvate through glycolysis, the metabolism of ketone bodies does not require the use of ATP [4]. The reactions involved in the production and oxidation of ketone bodies are illustrated in Figure 1.

### Excretion of ketone bodies

Both AcAc and βHB are excreted by the kidneys and, notably, their reabsorption is increased in extended starvation. Importantly, although ketones are indeed acidic, acid-base disturbances in diabetic ketoacidosis are not caused by ketone body accumulation (in fact, their excretion is increased), but by volume contraction and changes in bicarbonate and electrolyte concentrations [14,15].

### Regulation of the production and oxidation of ketone bodies

Ketogenesis occurs in response to metabolic demand for oxidative fuel when carbohydrate stores are low, a state communicated to adipocytes as a low insulin, high cortisol, high glucagon state. βHB also auto-regulates its own production by inhibiting lipolysis via PUMA-G nicotinic receptors on adipocytes [16] and decreasing free-fatty acid availability for ketogenesis. Within hepatocytes, ketogenic rates are fine-tuned, not only by fatty acid flux and concentrations of acetyl-CoA, but also by deacetylating mitochondrial 3-hydroxy-3-methylglutaryl CoA synthase 2, a rate-limiting enzyme in ketogenesis, via SIRT3 [17].

It is worth noticing that the conversion of AcAc into βHB, or vice versa, occurs in an NAD^+^/NADH-coupled near-equilibrium reaction and that the ratio of AcAc/βHB is proportional to the mitochondrial NAD^+^/NADH ratio. In other words, the activity of βHB dehydrogenase modulates the mitochondrial redox potential and ensures ketone bodies are terminally oxidised in proportion to the cellular ATP demand [18]. Furthermore, because the MCT are H^+^-coupled transporters, the NAD^+/^NADH ratio in the target tissues influences cellular uptake and oxidation proportionally to their energetic needs [11].

## The βHB monoester compared with other ketogenic interventions

As previously described, the production of ketone bodies is a dynamic process with multiple inputs and regulatory points. Therefore, is not possible to accurately set blood βHB concentrations with the interventions that rely on endogenous ketosis. The advent of exogenous sources of ketone bodies, such as the salts and esters, has enabled the accurate manipulation of ketone body blood concentration and introduced an entirely new metabolic state: exogenous ketosis [19]. However, achieving a fasting-like concentration of blood βHB with ketone salts would require the consumption of many grams of salt. Additionally, most salts are racemic, with 50% the bioactive d-form of βHB and 50% the l-form of βHB [20], which is not normally found in blood. Using doses between 357 and 714 mg/kg, the βHB monoester allows the safe and accurate attainment of blood concentrations of βHB similar to those observed after several days of fasting, but within 30 min [21]. Moreover, it delivers only the d-form of βHB and is salt-free.

A notable ‘middle point' between endogenous and exogenous ketogenic interventions is the consumption of medium-chain triglycerides (MCTS) [22]. After ingestion, MCTS are rapidly taken up in the portal system and converted into ketone bodies in hepatocytes. Simply, they are an exogenous ketogenic precursor converted into ketones by normal endogenous means. While they indeed acutely raise βHB blood concentration, they do so moderately and do not permit the accurate titration of βHB concentration. One thing MCTS and exogenous ketones have in common is that their consumption may induce gastrointestinal discomfort. However, the frequency and severity of these symptoms are dose- and compound-specific [23]. Table 1 summarises the differences between available ketogenic interventions.

**Table 1.** Endogenous and exogenous sources of the ketone bodies, d-β-hydroxybutyrate and acetoacetate, their ratios, the maximum blood βHB concentrations that can be reached, plus the insulin and glucose concentrations observed in their presence [1,19,22]

	Fasting	Ketogenic diet MCTS	Ketone salts	βHB monoester
Ketone source	Liver	Liver	Salt form of βHB	(*R*)-3-hydroxybutyl (*R*)-3-hydroxybutyrate
βHB:AcAc	5 : 1	3 : 1	3 : 1	5 : 1
Blood βHB (mM)	6–7	1–4	1–2	3–6
Insulin	Low	Low	Normal	Normal
Blood glucose	Low	Low	Low-Normal	Low-Normal

## The metabolism of the βHB monoester

**Figure 2.**
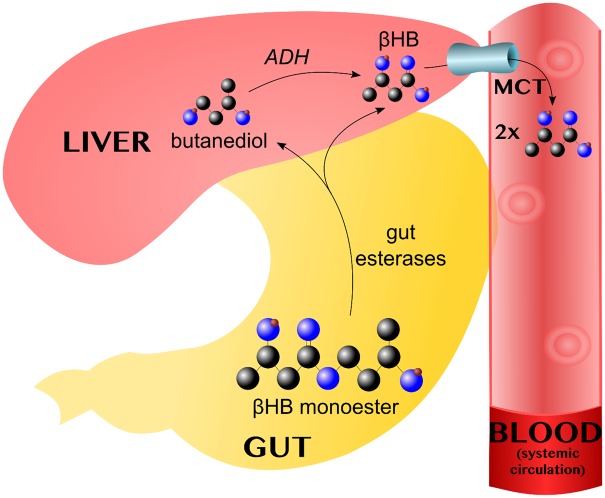
The βHB monoester bond is cleaved by gut esterases, yielding βHB and butanediol, which enter the portal circulation. In the liver, alcohol dehydrogenase (ADH) converts butanediol converted into βHB, which leaves via the monocarboxylate transporters (MCT). Each monoester molecule thus yields two βHB equivalents.

After ingestion, the βHB monoester bond is cleaved by esterases in the gut wall, yielding βHB and butanediol in equal amounts. Both are absorbed into the portal circulation and the latter is taken up by the liver, where it is converted into βHB by alcohol dehydrogenase (ADH). βHB leaves the hepatocytes via the MCT. Pharmacokinetic studies have shown that, in the fasted and resting states, the βHB monoester can induce ketoses for 3–4 h, with a peak at ∼1 h that is dose-dependent [21]. The metabolism of the βHB monoester is summarised in Figure 2.

## The potential applications of induced ketosis

To date, most of the studies involving the βHB monoester in humans have focused on exercise in athletes, but the rationale behind this approach is outside the scope of this review [24]. Although ketogenic diets and intermittent fasting strategies for metabolic health have regained popularity in the last few years [25], research on the mechanisms behind their benefits is scarce and/or difficult to interpret because the interventions result in many physiological changes and because of the inherent limitations of animal models and diet studies [26]. In this context, the βHB monoester offers the possibility of consistently, accurately, and singularly inducing high βHB blood concentrations in humans to allow the determination of results due to ketosis itself.

Arguably, the most exciting applications of the βHB monoester are therapeutic because many diseases are caused or exacerbated by energy deficits or abnormal metabolic regulation, thus inducing ketosis may be beneficial [4]. In the following section, we summarise the biochemical and physiological rationale behind the βHB monoester's use in specific diseases.

### Refractory epilepsy

Ketogenic diets have been used as a treatment for epilepsy for centuries [27]. Although the benefits are clear [28], the diets are far from ideal because they have been associated with an increased risk of nephrolithiasis [29], stunted growth (due to the low insulin concentration) and poor compliance, the diets proving difficult for many patients and their parents [30].

There is still some debate on whether the mechanism for the ketogenic diet effect is due to the elevated ketone bodies themselves. Mouse studies have suggested that changes in fat metabolism and/or the composition of the microbiome may underly the benefits [31]. On the other hand, there is compelling evidence supporting the utilisation of ketone supplements in other neuro-excitability related illnesses, such as schizophrenia [32] and migraine [33] by modifying local neurotransmitter systems and reducing local inflammation. To our knowledge, there haven't been yet long-term studies using any form of exogenous ketones as an adjuvant treatment for refractory epilepsy.

### Neurodegenerative diseases

Neurodegenerative diseases are multifactorial with complex underlying pathophysiological mechanisms. Not surprisingly, therefore, the standard monotherapeutic drug approach to conditions such as Alzheimer's and Parkinson's diseases has consistently failed to produce therapeutic or preventative treatments.

By addressing several of the key pathological mechanisms underlying neurodegenerative diseases simultaneously, ketogenic nutrition, including using a βHB monoester drink, could slow or halt the progression of conditions such as Alzheimer's and Parkinson's diseases. A more comprehensive and detailed review can be found elsewhere [34]; however, we will briefly, summarise some of the most relevant mechanisms here:

First, neurodegenerative diseases are marked by defects in mitochondrial metabolism, such as decreased ATP production, increase ROS production, complex IV dysfunction in Alzheimer's disease and complex I dysfunction in Parkinson's disease [35–38]. Ketones may improve mitochondrial metabolism via a number of mechanisms, including, but not limited to, improving the health of the mitochondrial pool by inducing mitochondrial turnover (inducing mitophagy and PGC-1α) in combination with reducing oxidative stress (decreasing ROS production and increasing antioxidant defences) and damage to mitochondrial DNA and proteins, as well as by simply serving as an alternative energy substrate where glucose metabolism is impaired [39–41].

Second, neurodegenerative diseases almost universally feature neuroinflammation. By inhibiting pathologic microglial activation [42] and key regulators in inflammation pathways, such as the NLRP3 inflammasome [7], a βHB monoester could also protect against neuroinflammation.

Third, ketones can protect against the toxicity of neurotoxins, such as Aβ oligomers and MPP+ in models of Alzheimer's and Parkinson's, respectively [43].

It is unlikely that a single intervention (either drug, diet or ketone supplement) can alone salvage or repair a severely diseased brain. However, by combining interventions, along with other lifestyle changes (sleep, exercise, stress reduction, etc.), it may be possible to develop a multifaceted approach to effectively treat these debilitating diseases. A study involving a monthly intervention of the βHB monoester in people living with Parkinson's disease is currently in progress [44].

### Heart failure

Today, cardiovascular disease is the most frequent cause of death [45]. Ketone oxidation improved hydraulic efficiency and ATP production in the working rat heart [3] and ketone body oxidation is increased in heart failure [46]; however, patients with heart failure rarely have a high enough βHB blood concentration to properly fuel their cardiac demands. A recent study in heart failure patients reported an acute 40% improvement in cardiac output and 8% improvement of left ventricular ejection fraction after infusion of βHB salts [47], yet salt consumption causes fluid retention and current therapeutic guidelines recommend a daily salt intake of less than 2 grams [48]. Consequently, a salt-based ketogenic supplement would be an unfeasible treatment for chronic heart failure. It is also worth mentioning that most current treatments for chronic heart failure are aimed at slowing its progression. Inotropic drugs, such as digoxin or levosimendan, are only prescribed in late stages of the disease or during acute exacerbations, respectively [48]. Therefore, any intervention that ameliorates a diminished cardiac output and can be consumed chronically could be highly desirable.

### Diabetes

In recent years, ketogenic diets have yielded positive results, to the point of reversing type 2 diabetes in 54% of patients in one 2-year study [49]. However, it is unclear if the benefit arose from a reduced glucose load or a ketone specific mechanism. It is an undisputed fact that, regardless of the animal model and regardless of the ketogenic intervention, acute ketosis lowers blood glucose within a few minutes [20,50,51]. There is evidence suggesting ketosis acutely reduces the glycaemic response to a glucose challenge [52], and improves insulin secretion [50] however, ketone infusions lower blood glucose concentration even in the absence of insulin [53] and there is current research looking into the effect of acute ketosis substrate availability for gluconeogenesis [54]. On the other hand, diabetes treatment involves many non-glucose related goals [55] and dyslipidaemia is one of the hallmarks of this disease. Additionally, most diabetes patients die from cardiovascular-related complications [56]. Therefore, the already described effects in cardiac output, and the added fact that acute ketosis also reduces free fatty acids [16,20], could prove beneficial for these patients. A study involving a monthly intervention with the βHB monoester in people living with type 2 diabetes is in progress [57].

### Sepsis related muscle atrophy

Arguably, ketone metabolism evolved to prevent muscle atrophy during prolonged catabolism [58] and the idea that inducing sustained ketosis in sepsis patients could ameliorate it has been around for at least three decades [59]. Sepsis is a hypercatabolic state usually worsened by undernutrition [60], and characterised by severe systemic inflammation [61]. On the other hand, ketone bodies themselves are a source of calories [62], βHB inhibits lipolysis via the PUMA-G receptor [16], reduces total energy expenditure by inhibiting short-chain fatty acid signalling through GPR41 [8] and most importantly, diminishes inflammation by blocking the NLRP3 inflammasome [7]. Enticing the idea of a similar outcome in sepsis patients, research on another hypercatabolic state: exercise [63], suggest ketone oxidation preserves glycogen, branched-chained amino acids, and total muscle mass [64]. Additionally, hyperglycaemia, is one of the most prominent hallmarks of sepsis [61] and is associated with mortality. By the same mechanisms already described, inducing ketosis could also help improve glucose control in sepsis patients. Despite this, the lack of safe exogenous precursors and significant gaps in our knowledge about its safety in this population have prevented further research. To our knowledge, the only study that has directly looked into it, involved a four-hour ketone salt infusion and showed improvements in glucose and fat metabolism but, understandably, was not long enough to show protein catabolism benefits [65]. Having the most thoroughly studied safety profile so far ketone supplement so far, the βHB monoester offers an opportunity to advance research on novel nutritional strategies for this critical condition.

## Conclusion

The βHB monoester is a promising treatment for of numerous diseases. However, more research is needed to describe its safety for specific patient populations, to assess the magnitude of its clinical benefits, and to increase our understanding about the underlying mechanisms behind its therapeutic effects. Additionally, allowing the quick and accurate manipulation of βHB concentration in the blood, it is a useful tool for ketone metabolism research.

## Perspectives

Since most pathologies are induced or aggravated by energy deficits or abnormal metabolic regulation, the induction of ketosis may be broadly beneficial. However, safety and accurately increasing blood βHB concentration is difficult with most ketogenic interventions, the exception being the βHB monoester.

- ***Importance***: Yielding only the d-isoform of βHB and being salt-free, the βHB monoester is a promising ketotherapeutic. Additionally, its pharmacological profile has been thoroughly studied.
- ***Current thinking***: The safety and tolerability of consuming the βHB monoester, three times per day for one month, has been demonstrated in healthy volunteers [66]; however, further disease-specific safety and tolerability studies are warranted. Safety and efficacy studies in type 2 diabetes mellitus [57] and Parkinson's disease [44] patients are in progress.
- ***Future directions***: As discussed, among the most exciting clinical applications of the βHB monoester are chronic diseases. However, due to its half-life, these applications would require patients to consume drinks frequently in order to sustain a fasting-like blood βHB concentration for a prolonged time. Additionally, the current price of chronically sustaining exogenous ketosis with the βHB monoester would impose an enormous financial burden for most patients and health systems. More research is needed to assess disease-specific cost-benefit ratios. Finally, despite the good tolerability reported in some studies, the bitter taste may impair adherence, particularly in children, the elderly and the critically ill.

## Abbreviations

AcAc: Acetoacetate
AcAc-CoA: Acetoacetyl-CoA
ACAT: Acetyl-coenzyme A acetyltransferases
ADH: alcohol dehydrogenase
ATP: Adenosine triphosphate
MCT: Monocarboxylate transporters
MCTS: Medium chain triglycerides
NAD: Nicotinamide adenine dinucleotide
NADH: Nicotinamide adenine dinucleotide + hydrogen (H)
ROS: Reactive oxygen species
SCOT: Succinyl-CoA-3-oxaloacid CoA transferase
βHB: d-β-hydroxybutyrate

## Open Access

Open access for this article was enabled by the participation of University of Oxford in an all-inclusive Read & Publish pilot with Portland Press and the Biochemical Society under a transformative agreement with JISC.
